# Supplementary material for: Precision fMRI reveals densely interdigitated network patches with conserved motifs in the lateral prefrontal cortex
Source: bioRxiv. 2025 Jul 30:2025.07.24.666468. Preprint. [Version 1] doi: 10.1101/2025.07.24.666468 (PMC12324188; doi:10.1101/2025.07.24.666468)
Supplement: Supplement 1 [file media-1.docx]

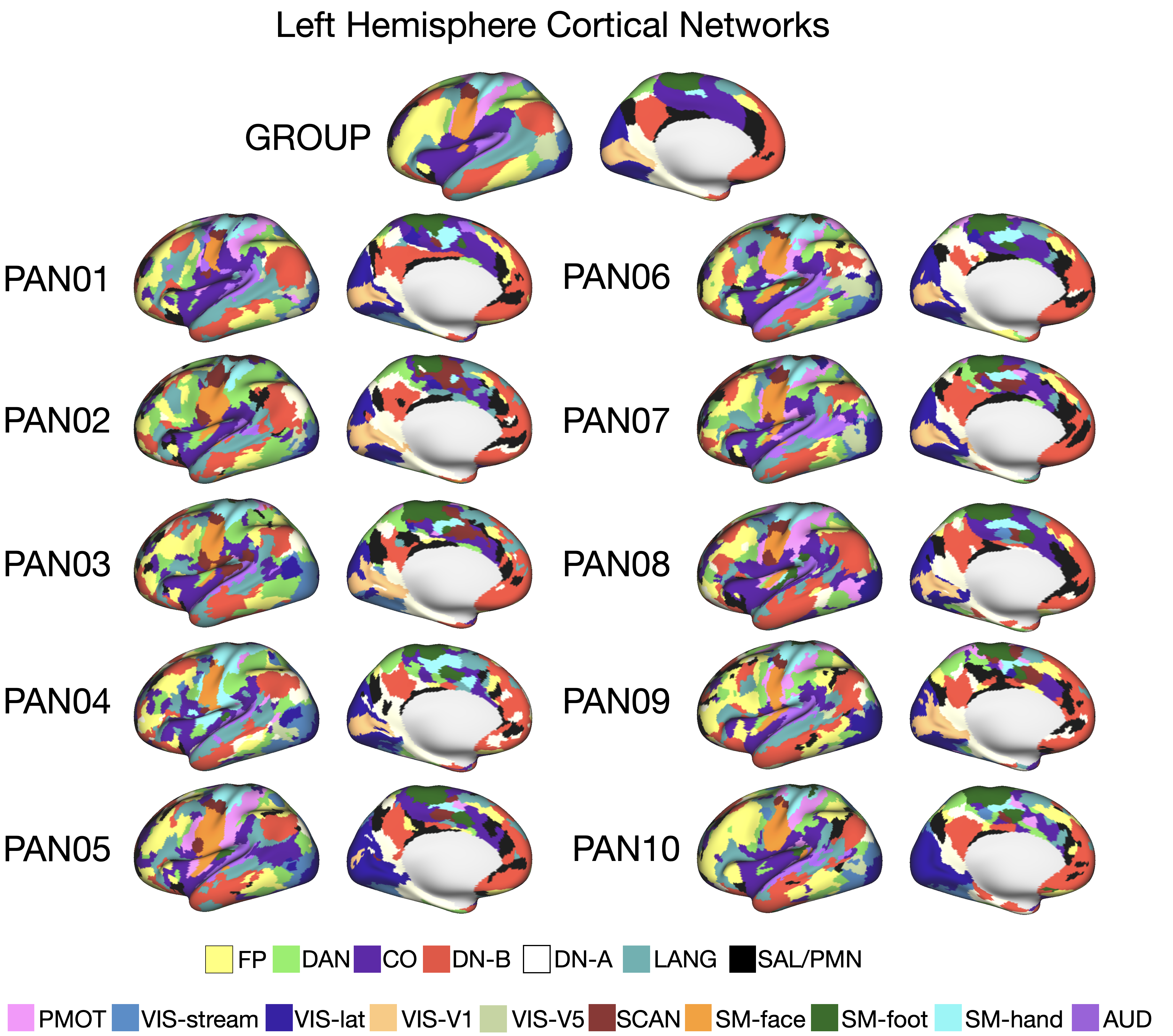


**Supplementary Figure S1.** **Individual-specific cortex-wide network parcellations (left hemisphere).**
Network parcellations were generated for all ten individuals using a cortex-wide network identification protocol adapted from Lynch et al. (2024). While the main text focuses on the lateral prefrontal cortex (LPFC), parcellations were defined across the entire cortical surface to ensure comprehensive coverage. Substantial individual variability in network topography is evident across the cortex.


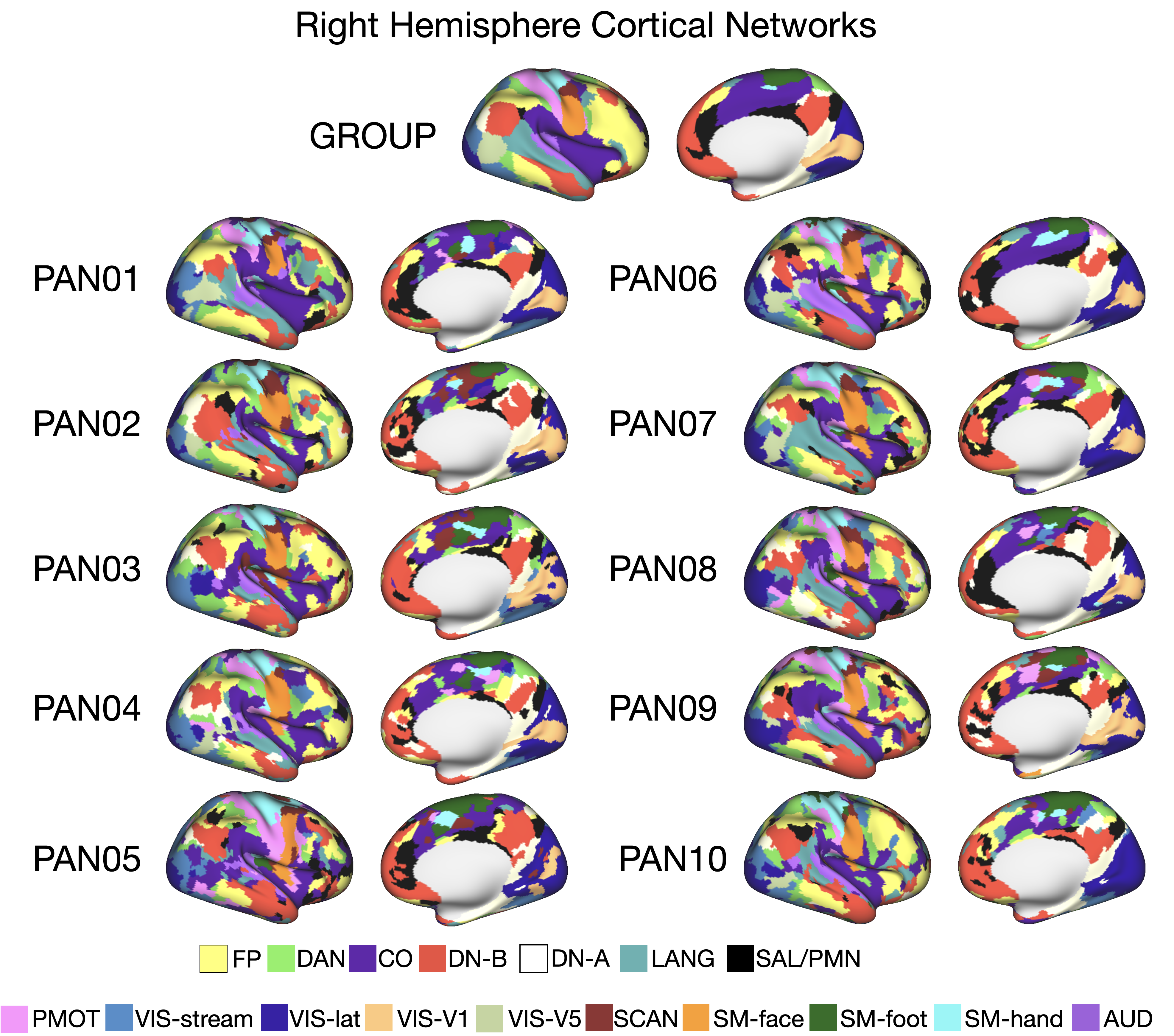


**Supplementary Figure S2.** **Individual-specific cortex-wide network parcellations (right hemisphere).**
Network parcellations were generated for all ten individuals using a cortex-wide network identification protocol adapted from Lynch et al. (2024). While the main text focuses on the lateral prefrontal cortex (LPFC), parcellations were defined across the entire cortical surface to ensure comprehensive coverage. Substantial individual variability in network topography is evident across the cortex.


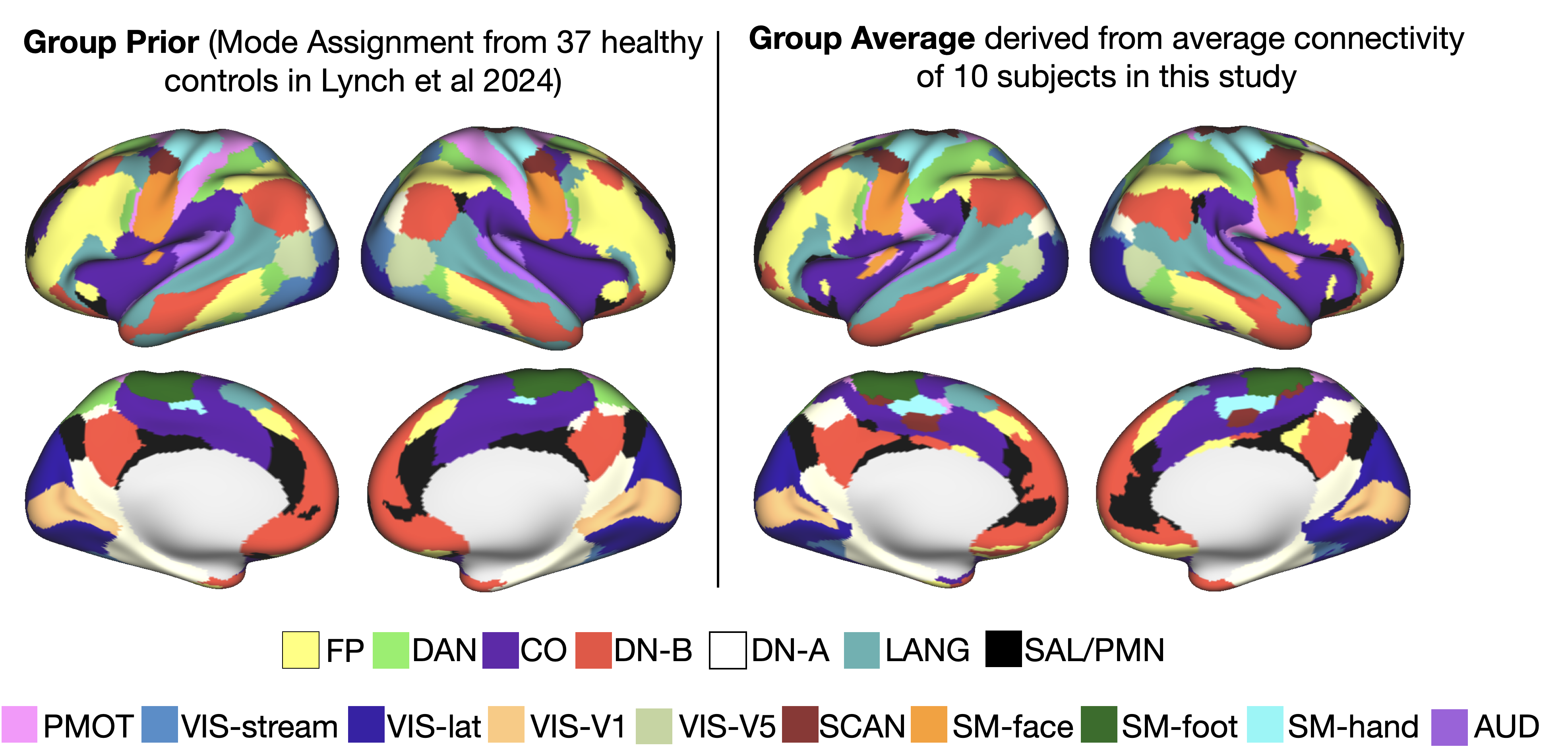


**Supplementary Figure S3.** **Group-average network priors are consistent across samples**.
To assess the robustness of group-average network structure, an alternative parcellation was generated using the same network identification as for individuals but using the average functional connectivity matrix from the ten individuals in the present study. This was visually compared to the mode parcellation derived from 37 healthy adults reported in Lynch et al. (2024). The resulting network maps were similar, indicating that the group-average is stable across independent samples and derivation methods.

**
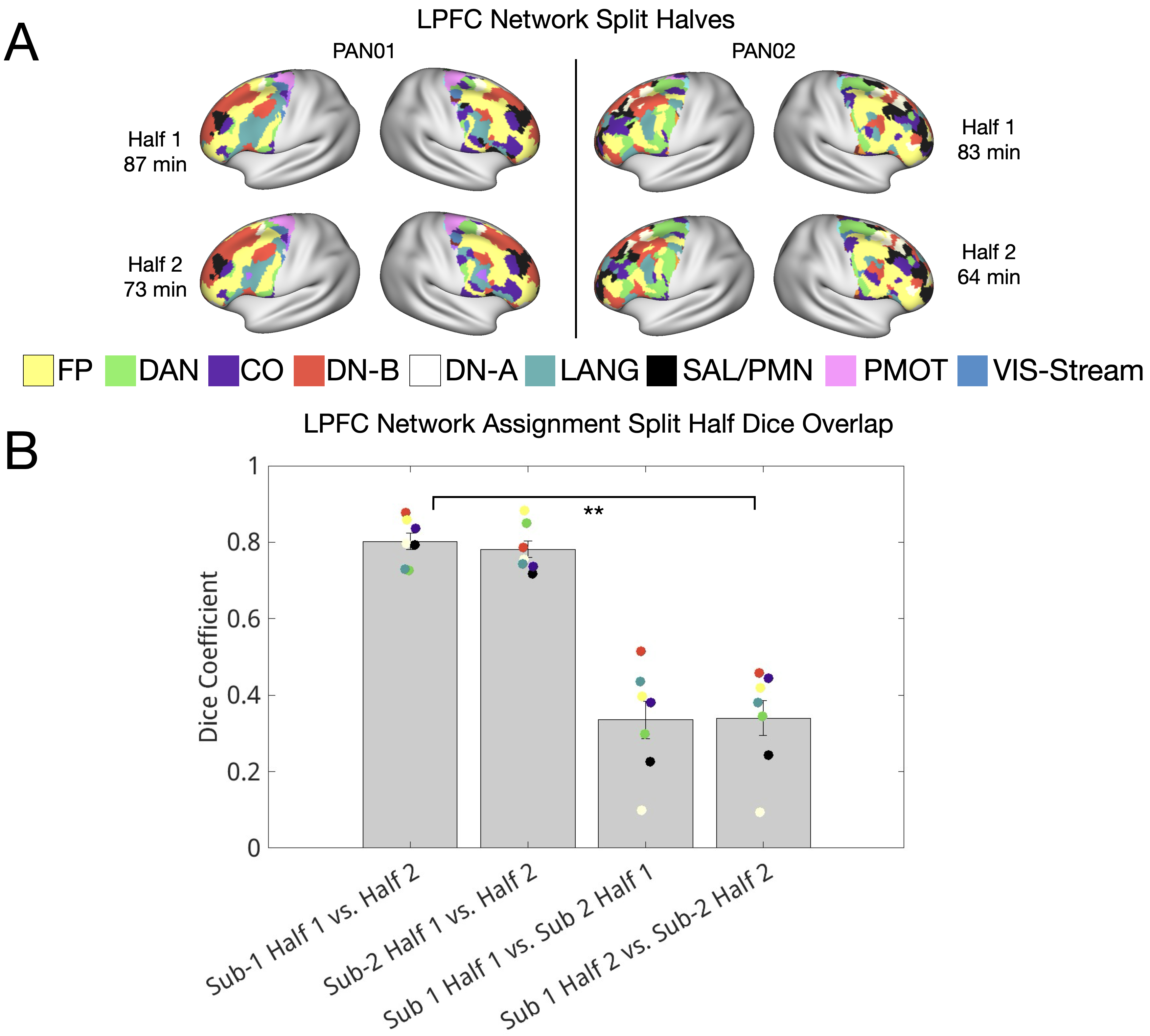
Supplementary Figure S4.** **LPFC network assignments are reliable within individuals across sessions and distinct across individuals.**
For two high-data individuals (PAN01 and PAN02), resting-state fMRI data were divided into two independent split-halves based on scanning day (odd vs. even sessions). Network parcellations were generated separately for each half using the same identification protocol. LPFC network topographies were highly similar across halves within individuals (mean Dice coefficient = 0.79 ± 0.05) but dissimilar between individuals (mean Dice = 0.34 ± 0.13). As indicated by an asterisk, a paired-samples t-test confirmed that within-individual similarity was significantly greater than between-individual similarity (t(6) = 9.8, p = 0.0001, Cohen’s d = 3.7), indicating robust within-subject reliability and pronounced individual specificity.


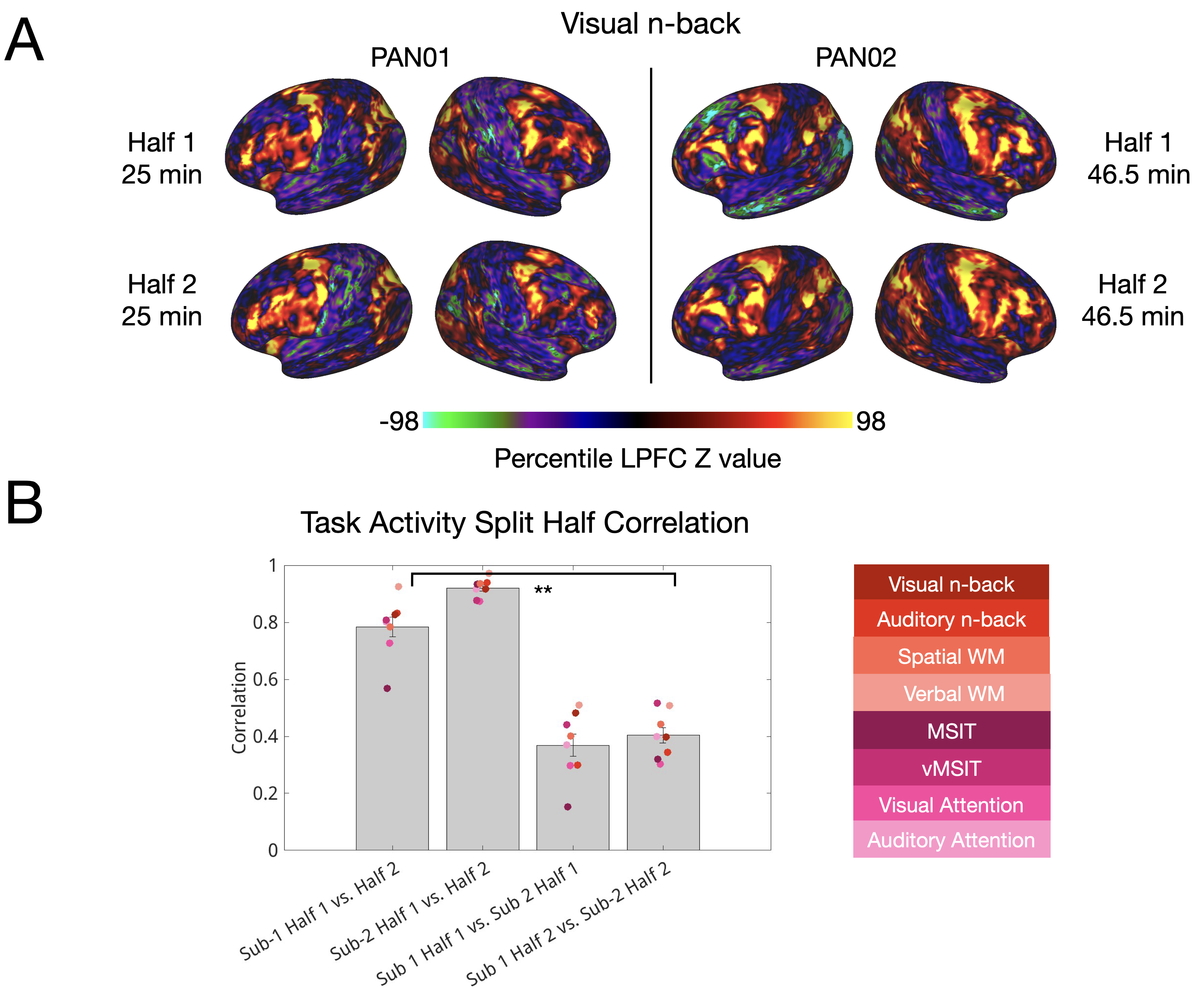


**Supplementary Figure S5.** **LPFC task activation patterns are reliable within individuals and distinct across individuals.**
For two high-data individuals (PAN01 and PAN02), task fMRI data were divided into two independent split-halves based on scanning day (odd vs. even sessions). Z-statistic maps were computed separately for each split-half. Task-evoked activation patterns in the lateral prefrontal cortex (LPFC) were highly consistent within individuals (mean correlation = 0.85 ± 0.06) but less similar between individuals (mean correlation = 0.39 ± 0.09). As indicated by an asterisk, a paired-samples t-test confirmed that within-individual similarity exceeded between-individual similarity (t(7) = 21.6, p = 0.000001, Cohen’s d = 7.6), highlighting the reliability and idiosyncrasy of individual activation profiles.


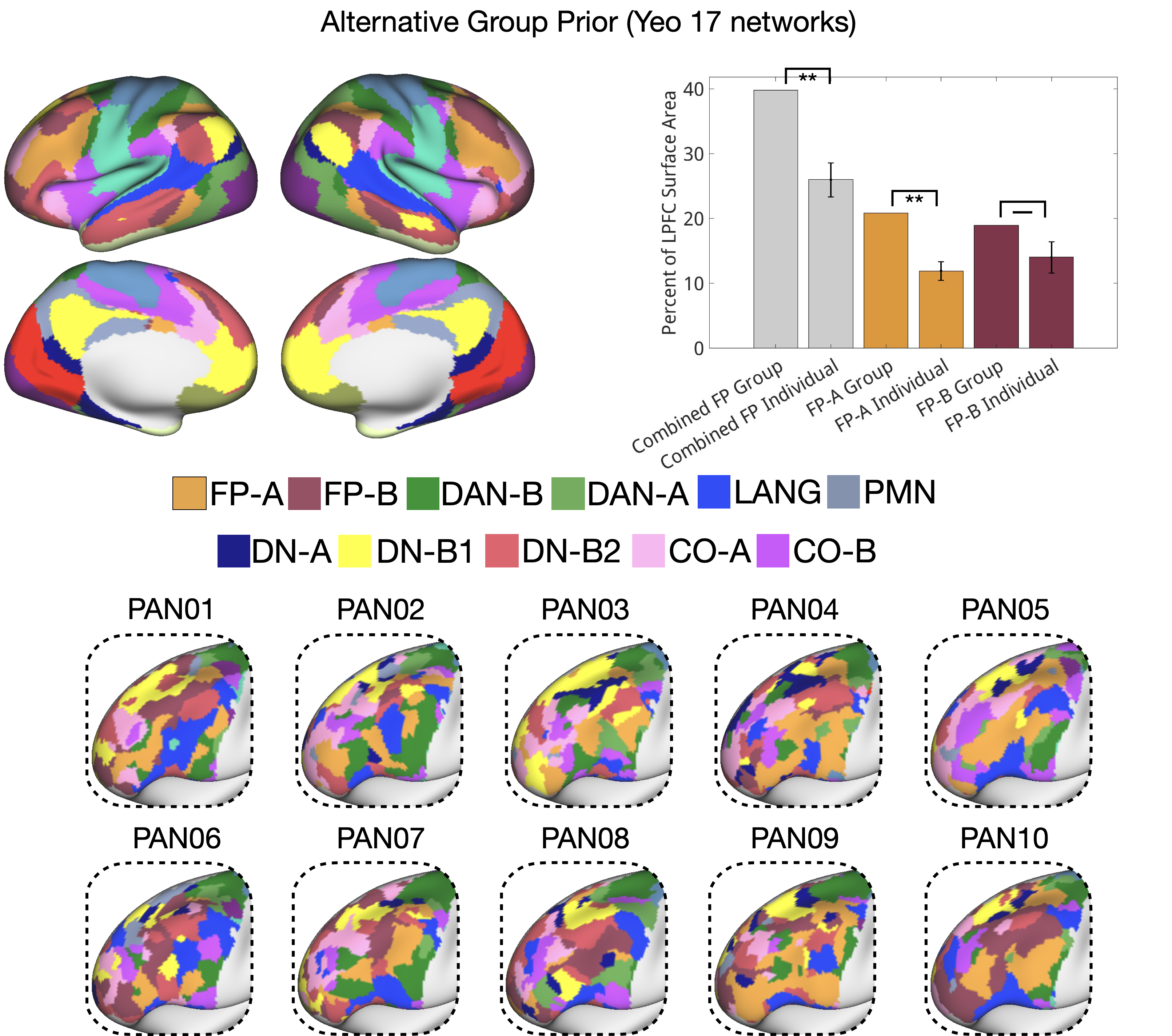


**Supplementary Figure S6.** **Replication of network composition results using an alternative group prior with two frontoparietal networks.**
To assess the robustness of our findings, individual-specific LPFC parcellations were re-derived using an alternative group prior that included two distinct frontoparietal networks (Yeo 17 networks, FP-A and FP-B; Yeo et al., 2011). The combined frontoparietal territory (FP-A + FP-B) occupied significantly more LPFC surface area in the group prior than in individual-specific parcellations (paired t-test: t(9) = 5.3, p = 0.0005, Cohen’s d = 1.68), replicating the primary finding of inflated frontoparietal representation in group-level maps. When examined separately, FP-A was significantly larger in the group prior (paired t-test: t(9) = 6.20, p = 0.0001, Cohen’s d = 1.96), while FP-B showed a non-significant trend in the same direction (paired t-test: t(9) = 2.04, p = 0.07, Cohen’s d = 0.64). Significant (p<0.05) differences are indicated with asterisks.

**
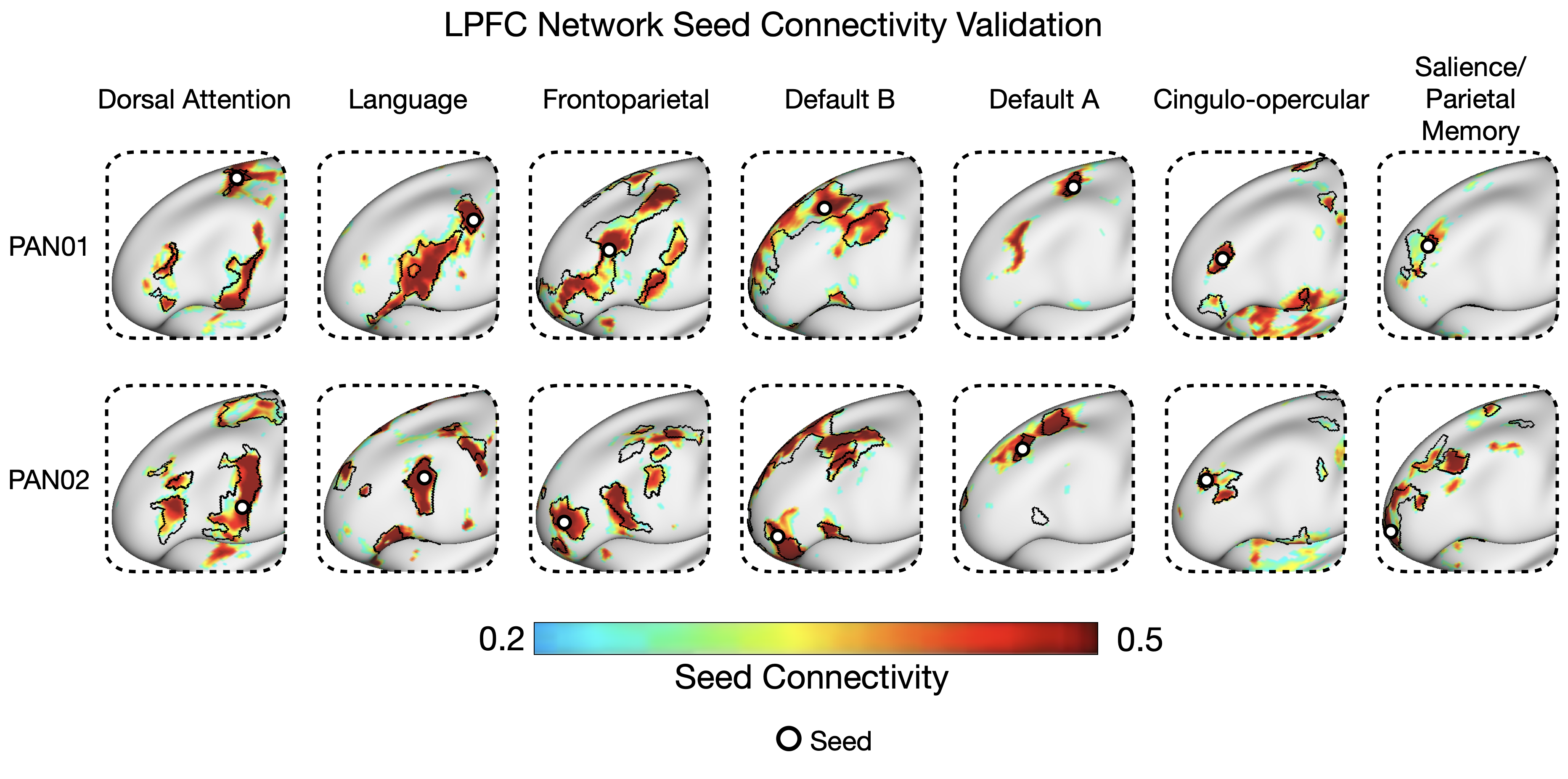
Supplementary Figure S7.** **LPFC network parcellations are validated by seed-based functional connectivity.**
To validate the fine-grained organization observed in LPFC network parcellations, seed-based connectivity maps were generated for two exemplar individuals (PAN01 and PAN02). Seeds were manually placed within the boundaries of each association network in the LPFC. The resulting connectivity maps closely matched the parcellated network territories, including the spatial patchiness observed relative to group-average maps, providing converging evidence for the reliability of individual-specific parcellations.

**
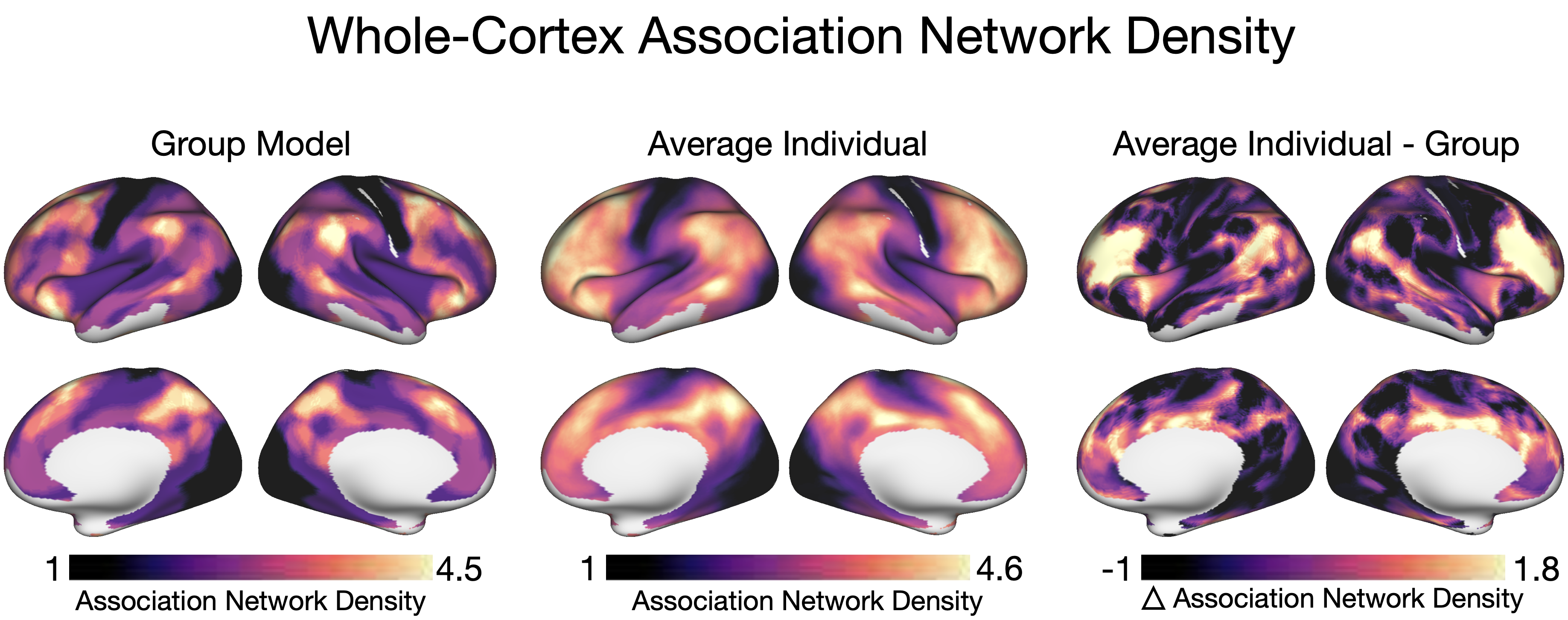
**

**Supplementary Figure S8.** **Association network density is underestimated by group averaging.**

Association network density is shown across the cortical surface for the group-average map (left), the across-individual average (middle), and the difference between the two (right). While broad patterns are generally preserved, group averaging underestimates association network density in the rostral LPFC.


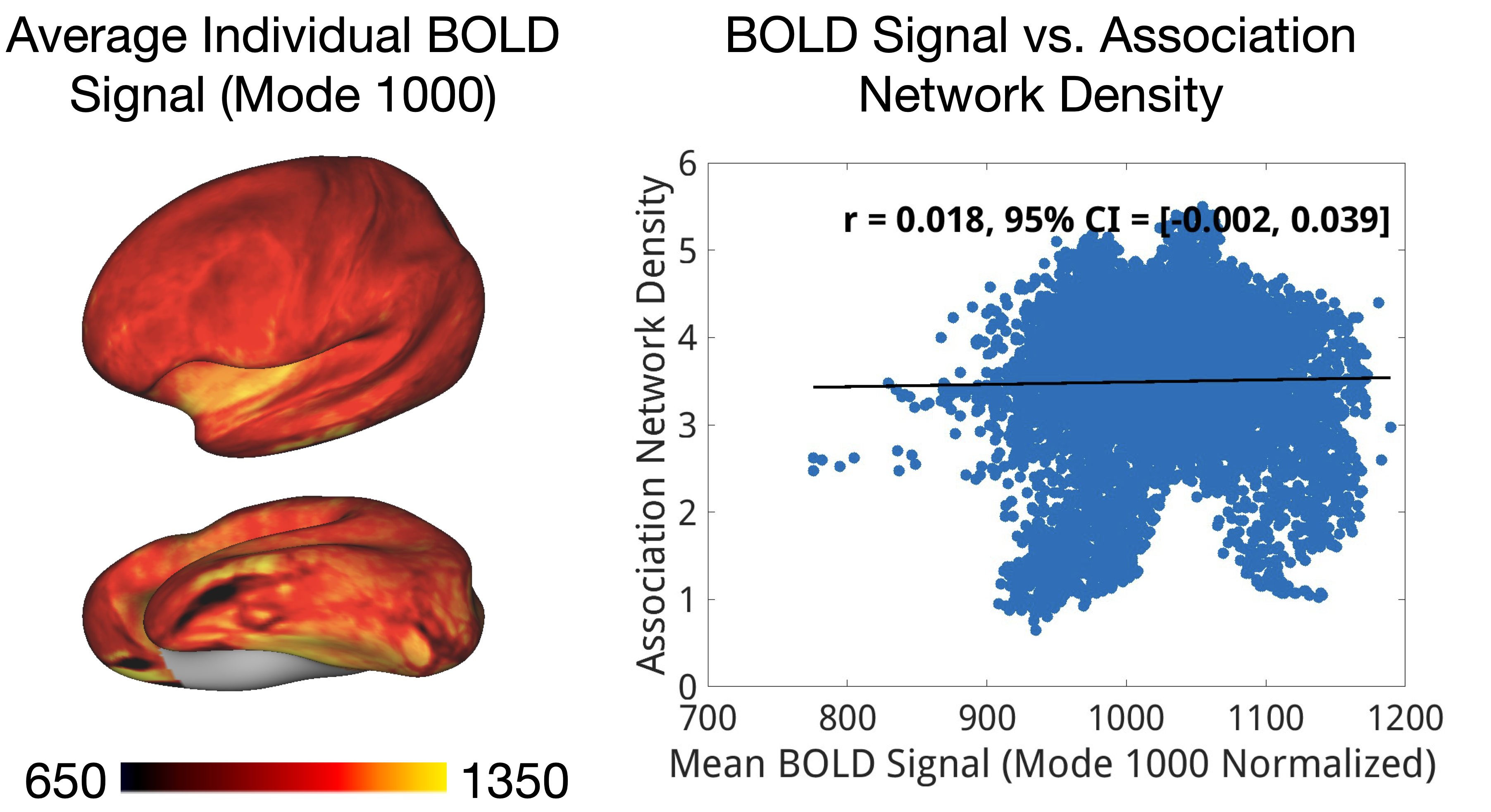


**Supplementary Figure S9. There is no meaningful relationship between association network density and BOLD signal in the LPFC.**

A measure of BOLD signal (after mode 1000 normalization) across individuals is shown across the left hemisphere cortical surface. Visually, there is no obvious pattern that corresponds with the high rostral association network density shown in Figure 2D and 2E. This is quantified (right) – the correlation between BOLD signal and association network density in the LPFC was near zero (r = 0.018, 95% CI: [–0.002, 0.039]), suggesting no meaningful relationship.

**
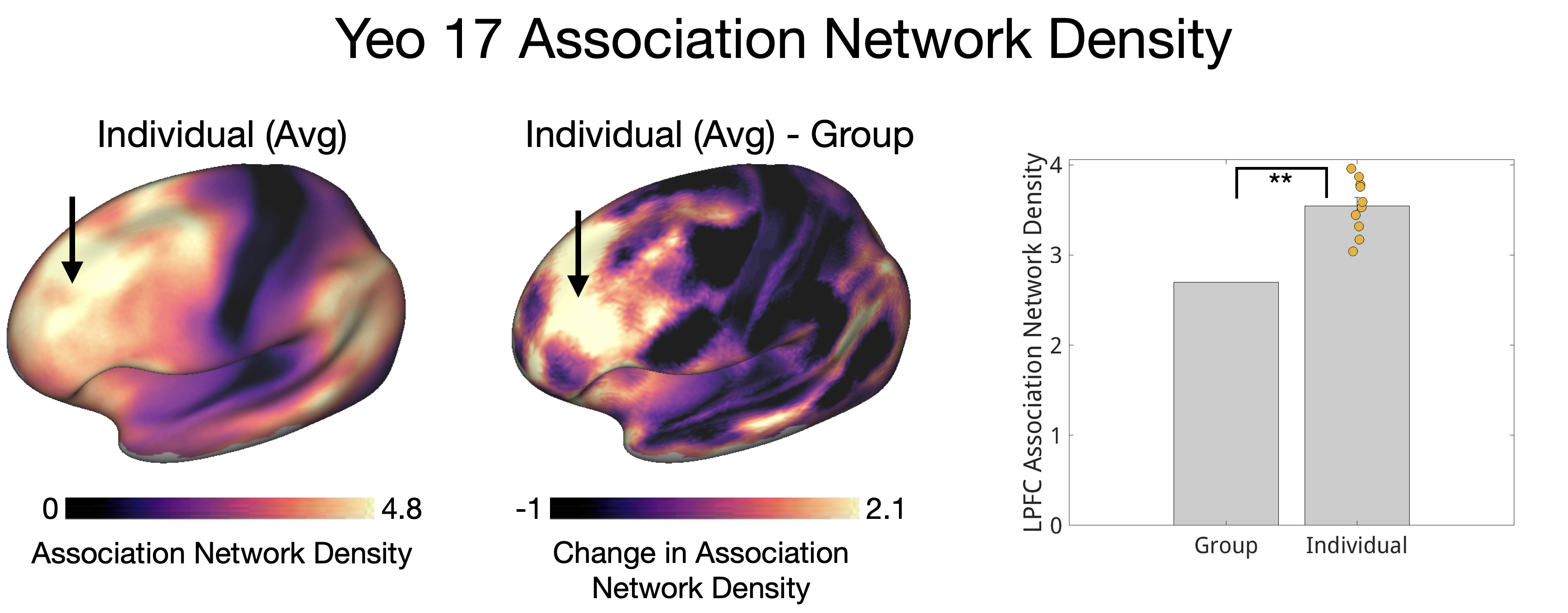
**

**Supplementary Figure S10.** **Replication of** **association network density using Yeo 17 group average atlas.**
Association network density is shown across the cortical surface for the across-individual average (left) and the difference between group-average and individual average (middle) using the Yeo 17 group average. Marked by an asterisk, individuals showed significantly higher association network density compared to the group average (paired t-test: t(9) = 8.82, p = 0.00001, Cohen’s d = 2.79)

**
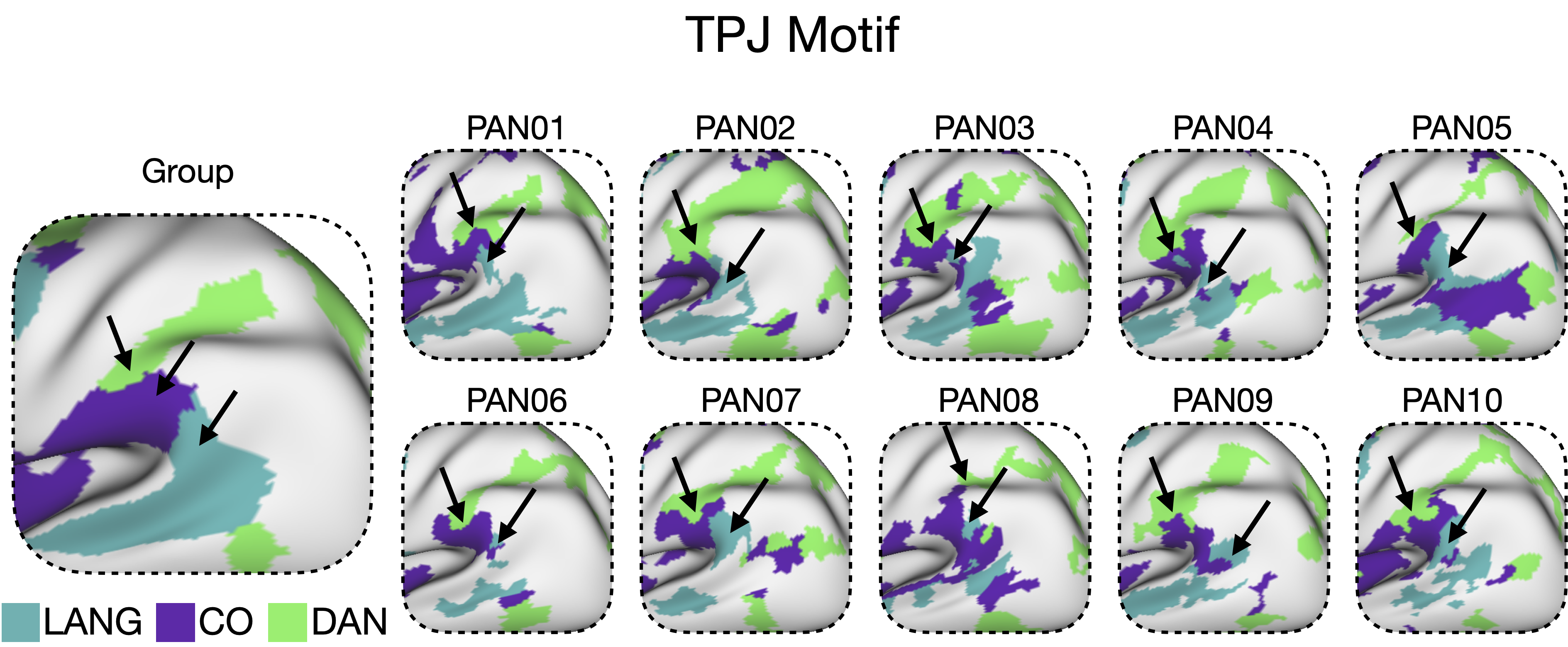
**

**
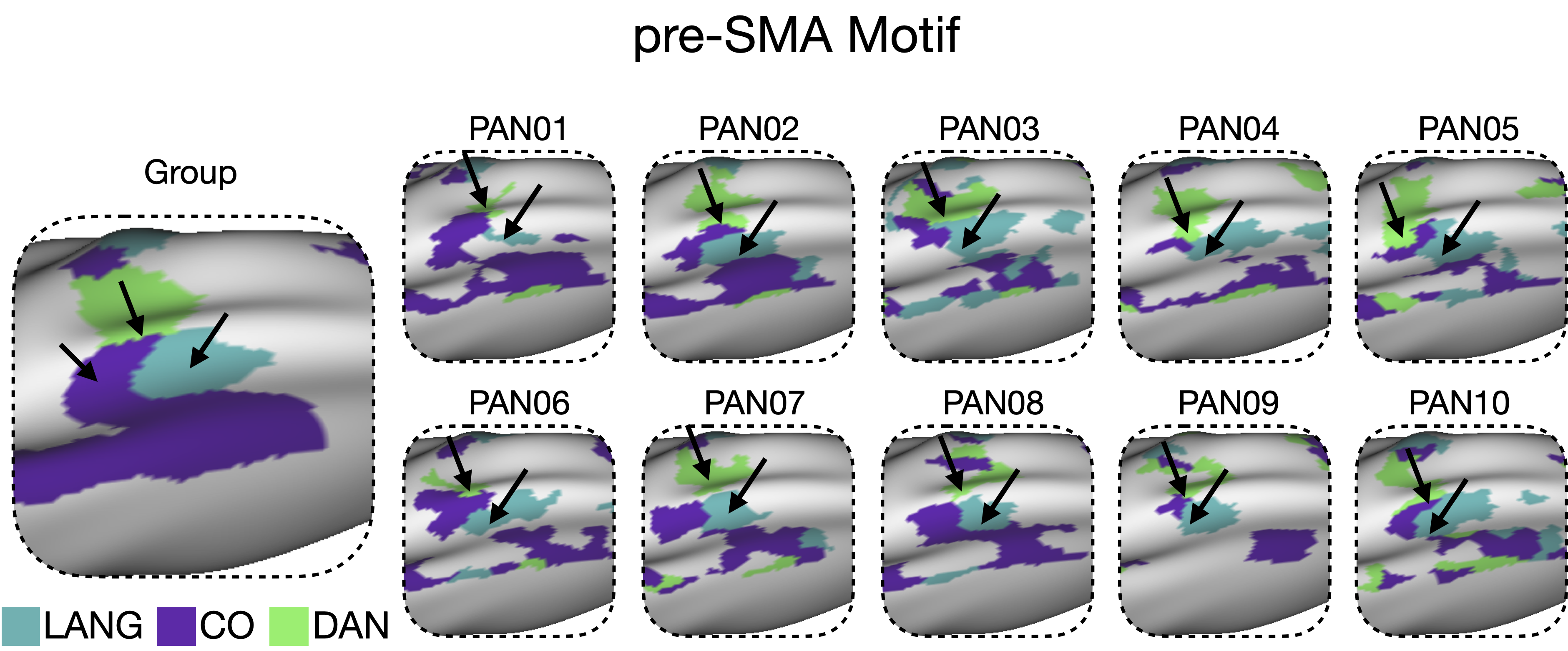
**

**Supplementary Figure S11.** **The DAN–CO–LANG motif recurs in both TPJ and pre-SMA across individuals.**
Network parcellations for each individual were manually examined for evidence of a repeating three-network motif involving the dorsal attention network (DAN), cingulo-opercular network (CO), and language network (LANG). This motif was observed in all ten individuals in both the temporoparietal junction (TPJ) and the pre-supplementary motor area (pre-SMA). In both regions, the spatial arrangement of the motif was sufficiently consistent across individuals that it is also evident in the group-average map.

**
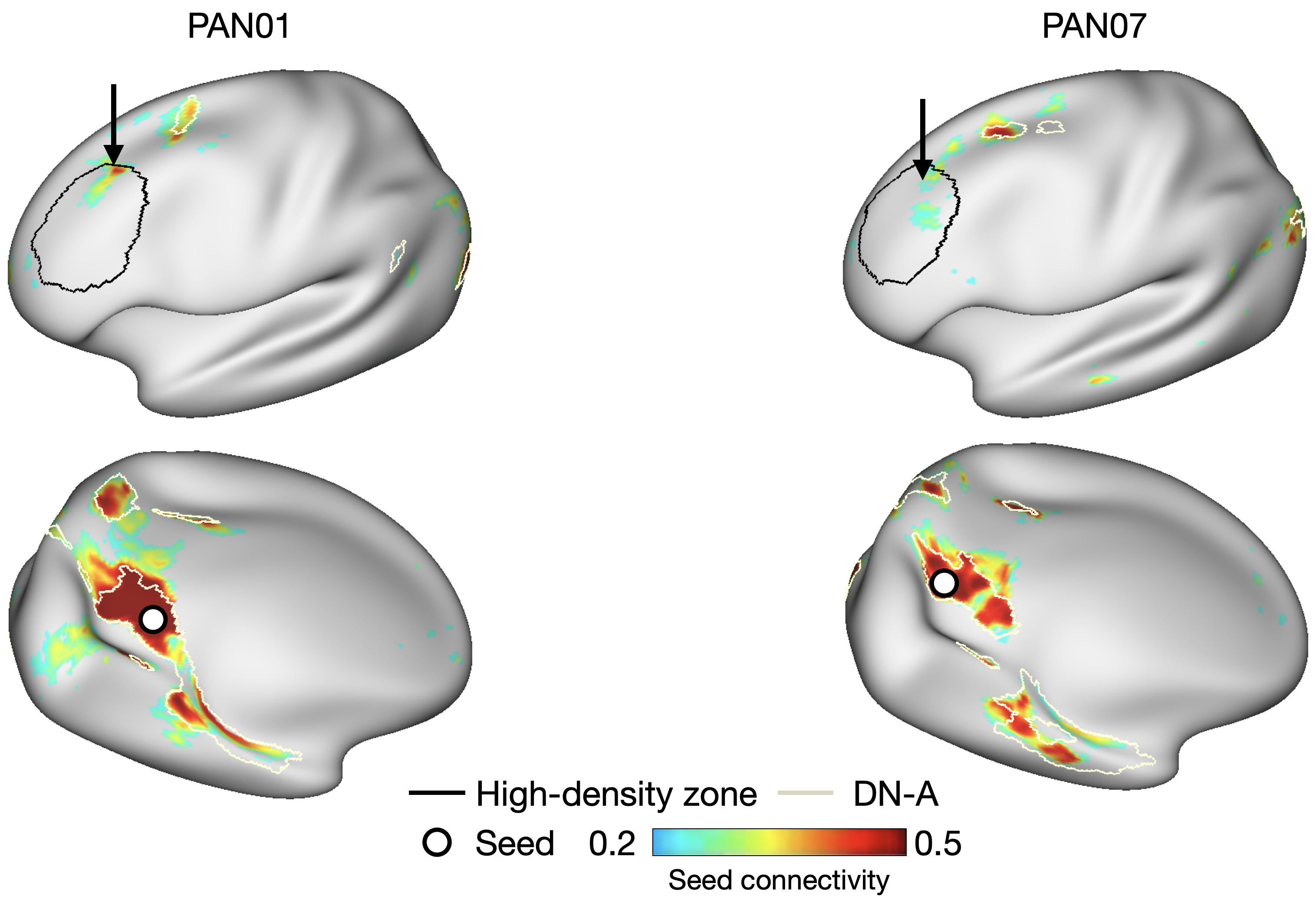
**

**Supplementary Figure S12.** **In exception cases, DN-A shows seed-based connectivity to the rostral high-density zone.**
PAN01 and PAN07 did not exhibit DN-A network territory within the rostral LPFC high-density zone based on their individual parcellations. However, manually selected seeds within DN-A in these individuals showed functional connectivity to this location. This suggests the possible presence of DN-A–related signal in this region that was not captured by the discrete parcellation boundaries. The high density zone shown is the same as in Figure 4 – a 15mm radius around the rostral CO region.


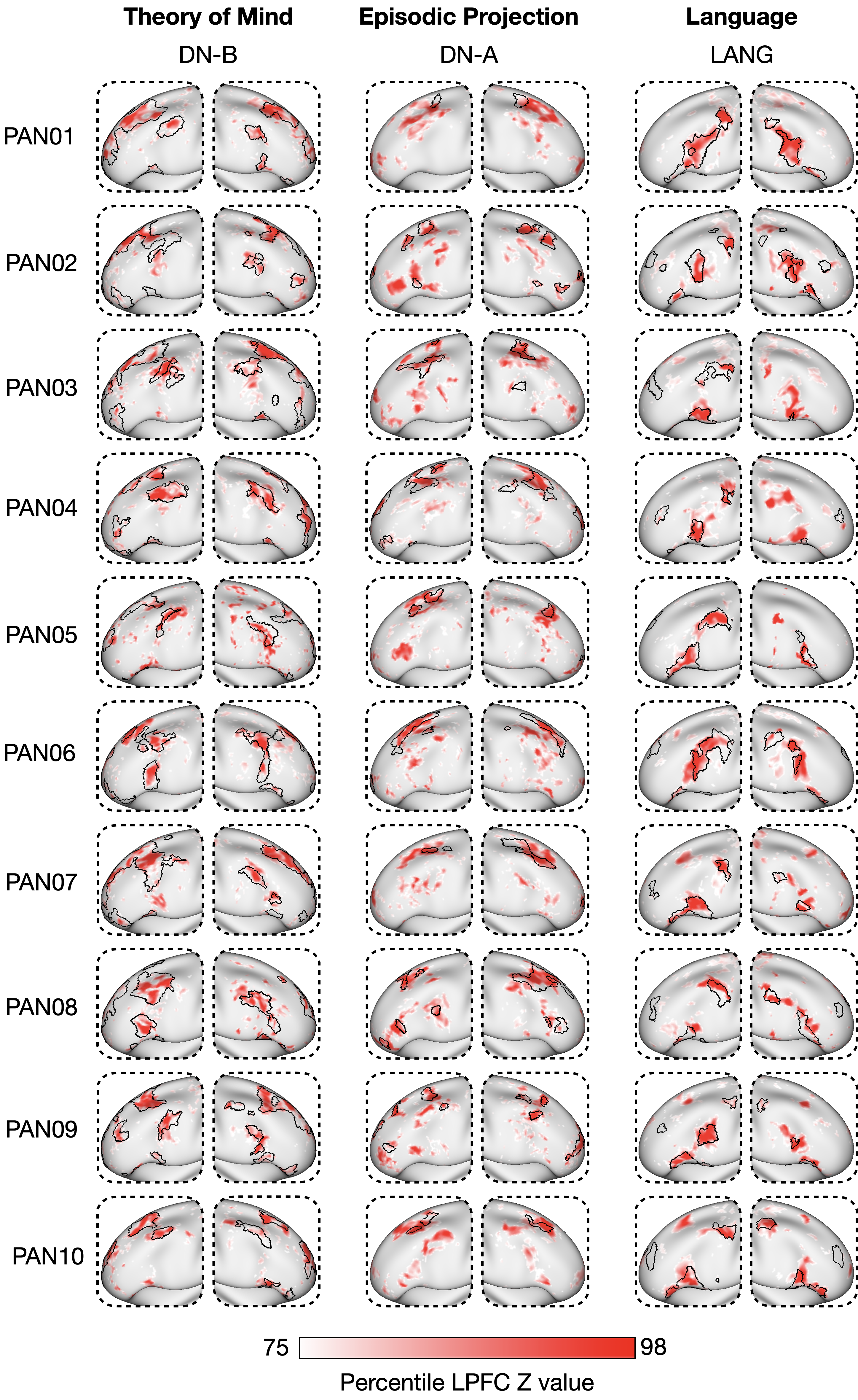


**Supplementary Figure S13.** **Theory of mind, episodic projection, and language task activation maps for all individuals.**
The top 25% most task-active LPFC vertices (ranked by z-statistic) are shown for all individuals for theory of mind, episodic projection, and language tasks, overlaid with individual-specific network borders for the relevant network (DN-B, DN-A, LANG).


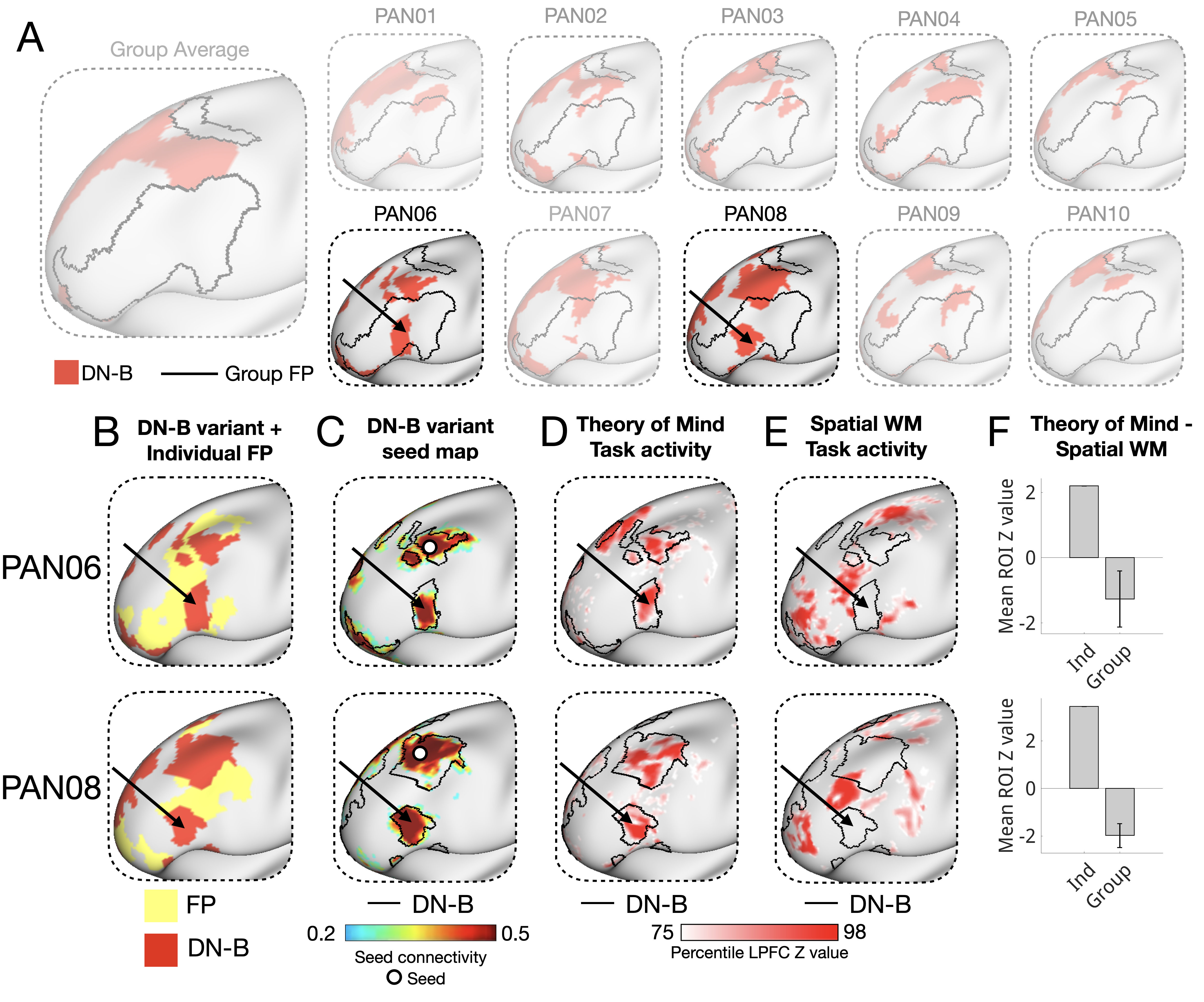


**Supplementary Figure S14. Idiosyncratic default B regions are embedded within canonical frontoparietal territory in a subset of individuals and are validated through multiple measures.**(A) Default B network parcellations for 10 individuals and the group average, overlaid with the group-average frontoparietal (FP) network. Two individuals (PAN06, PAN08) showed large default B regions in mid-LPFC, well outside typical default B territory. (B) These variant default B regions were interdigitated with individual-specific FP regions. (C) Seed-based connectivity from the variant regions showed coupling with canonical LPFC default B network regions, supporting their network identity. (D) Theory of mind task activations showed positive responses in the variant default B regions but not in adjacent FP regions. (E) Spatial working memory activations showed the opposite pattern: activation in adjacent FP regions but not in the variant default B regions. (F) Theory of mind > spatial working memory *z*-values are shown for the variant individuals and for the same ROI locations averaged across the eight non-variant individuals. Variant-region ROIs in non-variant individuals did not show theory of mind > spatial WM responses, confirming their individual-specific nature.

**
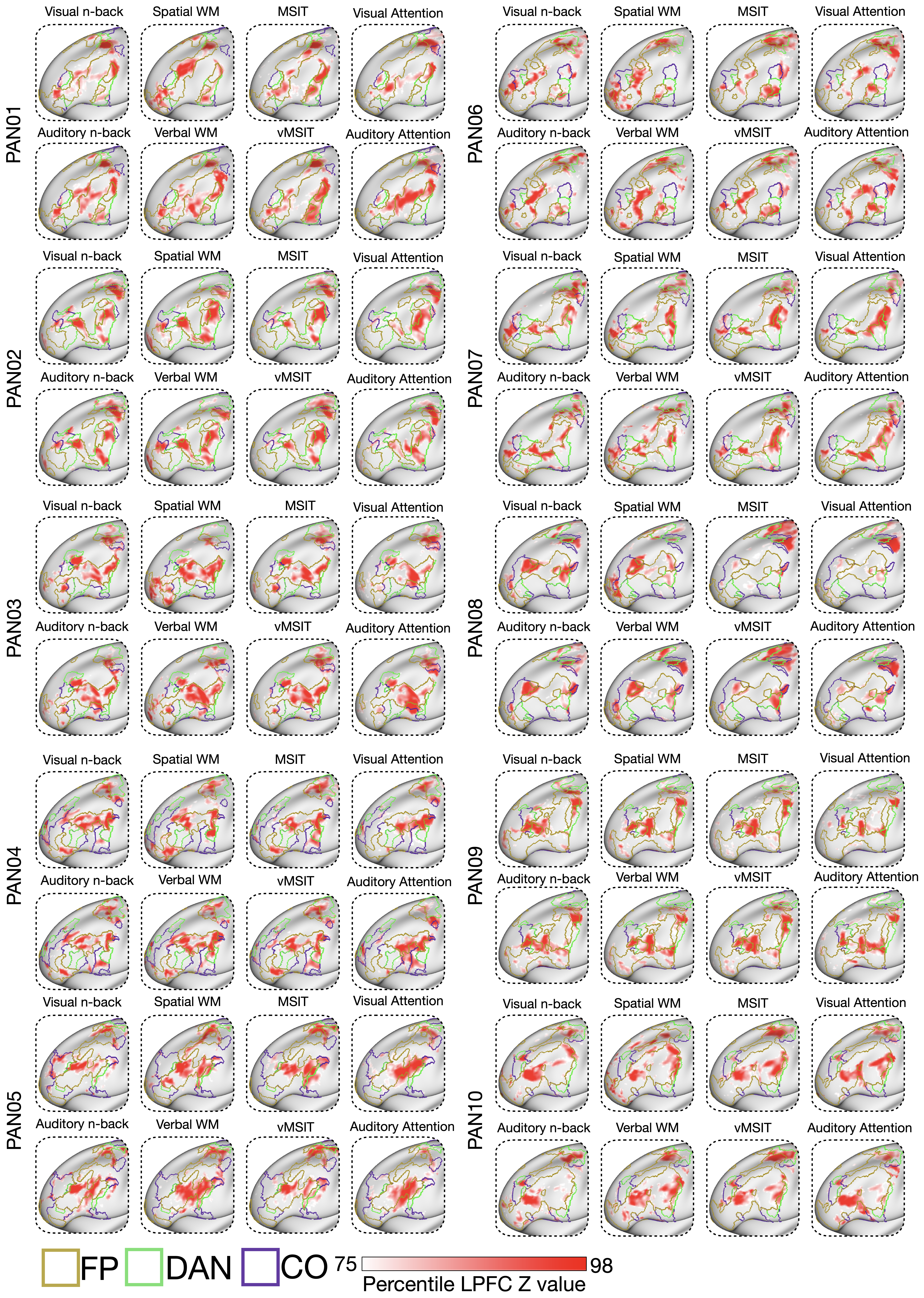
**

**Supplementary Figure S15. Left hemisphere cognitive control task activation maps for all individuals.**

Left hemisphere cognitive control task activation maps showing the top 25% activated LPFC vertices along with the outlines of the FP, CO, and DAN networks. As described in the main text, activation maps revealed a distributed set of highly active regions which were similar across the 8 different tasks, but unique between people. These regions tended to be located near or crossing the borders of the FP, CO, and DAN networks.


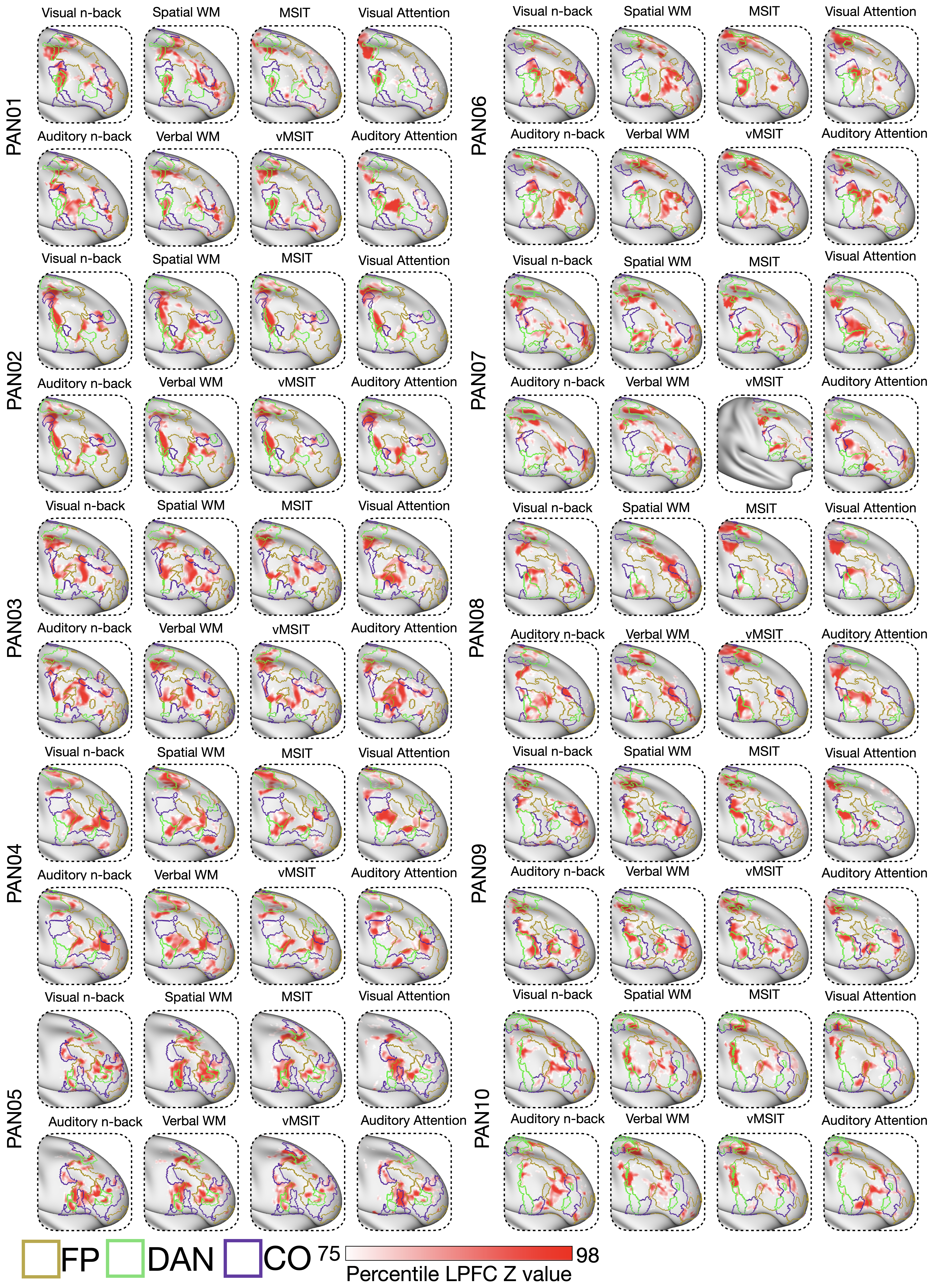


**Supplementary Figure S16.** **Right hemisphere cognitive control task activation maps for all individuals.**

Maps showing the top 25% activated LPFC right hemisphere vertices along with the outlines of the FP, CO, and DAN networks. As described in the main text, activation maps revealed a distributed set of highly active regions which were similar across the 8 different tasks, but unique between people. These regions tended to be located near or crossing the borders of the FP, CO, and DAN networks.

**
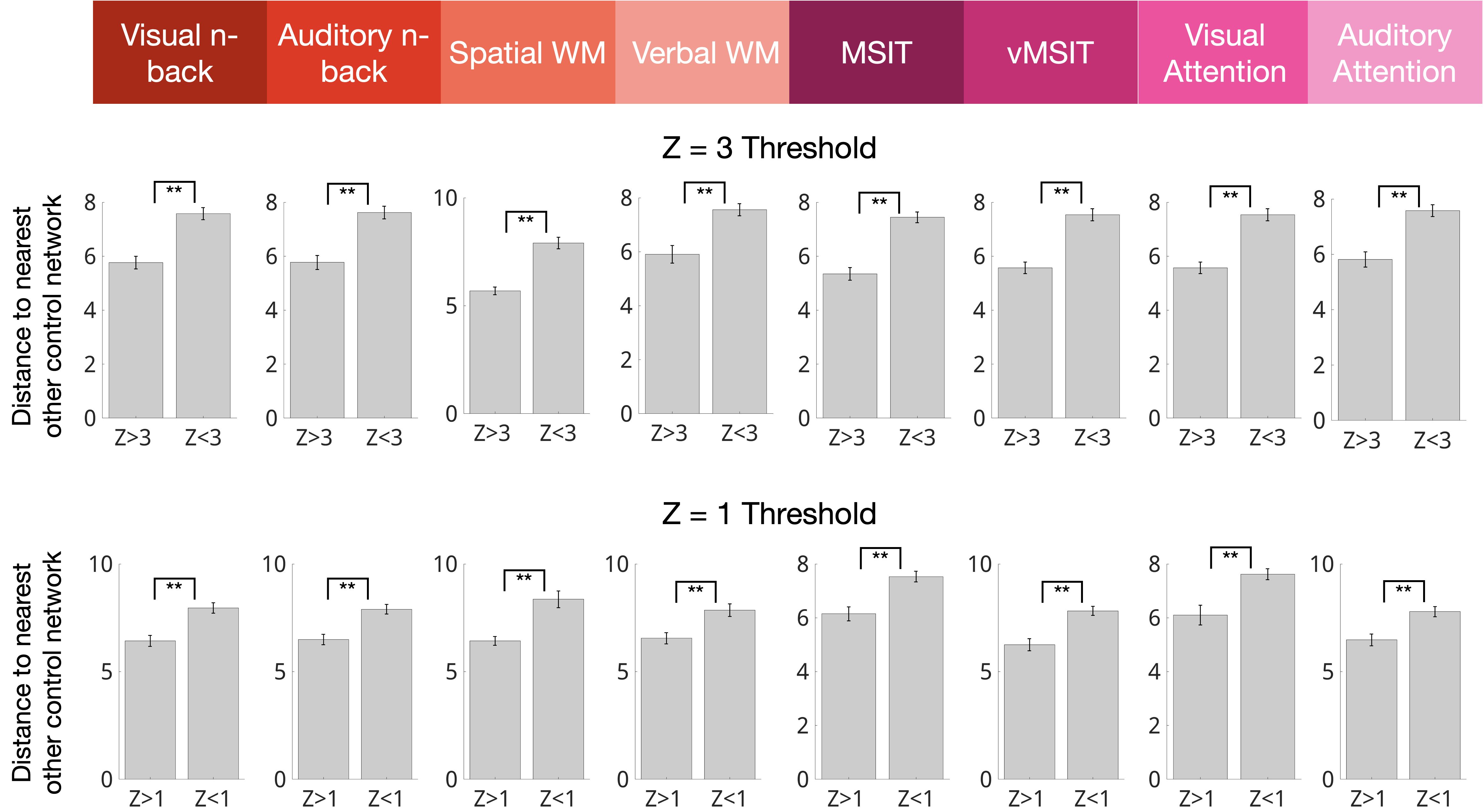
**

**Supplementary Figure S17.** **Cognitive control activations within control networks (FP, DAN, CO) tended to be near the borders where these networks meet.**

For each task, LPFC vertices in the FP, CO, and DAN networks were split into “active” (z > 3, z > 2, z > 1) or less-active (z < 3, z < 2, z < 1) groups, and the mean geodesic distance from those vertices to the other two control networks was calculated. Indicated by asterisks, the more active group was closer to the other two control networks (all p < 0.01) across all 8 tasks across all 3 thresholds. Z > 2 is shown in the main text.

Theory of Mind Comparisons (Target Network DN-B > Comparison Network)

| Comparison Network | Raw p-value | Corrected p-value (X6) | T-stats | Cohen’s d |
| --- | --- | --- | --- | --- |
| FP | 3.337e-05 | **0.0002002** | 6.9515 | 2.1983 |
| DAN | 9.2e-07 | **5.54e-06** | 10.822 | 3.4222 |
| CO | 5.58e-06 | **3.349e-05** | 8.7088 | 2.754 |
| DN-A | 0.00013899 | **0.00083392** | 5.7452 | 1.8168 |
| LANG | 0.00147005 | **0.00882029** | 4.0374 | 1.2767 |
| SAL/PMN | 7.23e-06 | **4.336e-05** | 8.4358 | 2.6676 |

Episodic Projection Comparisons (Target Network DN-A > Comparison Network)

| Comparison Network | Raw p-value | Corrected p-value (X6) | T-stats | Cohen’s d |
| --- | --- | --- | --- | --- |
| FP | 6.41e-06 | **3.845e-05** | 8.5619 | 2.7075 |
| DAN | 5.9e-07 | **3.57e-06** | 11.4015 | 3.6055 |
| CO | 1.9e-07 | **1.17e-06** | 12.9986 | 4.1105 |
| DN-B | 1.51e-06 | **9.07e-06** | 10.2045 | 3.2269 |
| LANG | 1.9e-07 | **1.12e-06** | 13.0632 | 4.131 |
| SAL/PMN | 1.6e-07 | **9.4e-07** | 13.3251 | 4.2138 |

Language Processing Comparisons (Target Network LANG > Comparison Network)

| Comparison | Raw p-value | Corrected p-value (X6) | T-stats | Cohen’s d |
| --- | --- | --- | --- | --- |
| FP | 8.05e-06 | **4.833e-05** | 8.3232 | 2.632 |
| DAN | 3.971e-05 | **0.00023826** | 6.7959 | 2.1491 |
| CO | 0.00011666 | **0.00069994** | 5.8852 | 1.8611 |
| DN-A | 8.18e-06 | **4.907e-05** | 8.3075 | 2.6271 |
| DN-B | 4.48e-06 | **2.689e-05** | 8.9465 | 2.8291 |
| SAL/PMN | 5.59e-06 | **3.355e-05** | 8.7069 | 2.7534 |

**Supplementary Table S1: Corrected and uncorrected p-values, t-stats and Cohen’s d for all network task activation comparisons to high level task domains.** Bolded corrected p-values are significant (corrected p < 0.05).

**Language Network/Language Task Overlap (Individual versus Comparison)**

| Comparison | Raw p-value | Corrected p-value (X2) | T-stats | Cohen’s d |
| --- | --- | --- | --- | --- |
| Group | 0.00603 | **0.0121** | 3.13 | 1.04 |
| Others | 0.000675 | **0.00135** | 4.57 | 1.52 |

**Default Network A /Episodic Projection Task Overlap**

| Comparison | Raw p-value | Corrected p-value (X2) | T-stats | Cohen’s d |
| --- | --- | --- | --- | --- |
| Group | 0.000865 | **0.00173** | 4.4 | 1.47 |
| Others | 0.0178 | **0.0357** | 2.47 | 0.823 |

**Default Network B /Theory of Mind Task Overlap**

| Comparison | Raw p-value | Corrected p-value (X2) | T-stats | Cohen’s d |
| --- | --- | --- | --- | --- |
| Group | 0.00068 | **0.00136** | 4.56 | 1.52 |
| Others | 1.9e-05 | **3.8e-05** | 7.47 | 2.49 |

**LPFC vs. Non-LPFC Network/Task Overlap**

| Network/Task | Raw p-value | Corrected p-value | T-stats | Cohen’s d |
| --- | --- | --- | --- | --- |
| Language/Language | 0.000148 | **0.000148** | 5.7 | 1.9 |
| DN-A/Episodic Projection | 0.0271 | **0.0271** | 2.21 | 0.738 |
| DN-B/Theory of Mind | 0.000307 | **0.000307** | 5.14 | 1.71 |
| Cross Task Average | 2.68e-05 | **2.68e-05** | 7.15 | 2.38 |

**Supplementary Table S2: Corrected and uncorrected p-values, t-stats and Cohen’s d for all network task activation overlap comparisons.** Bolded corrected p-values are significant (corrected p < 0.05).

| **Task** | **Network** | **Mean Z Value** | **T-stat** | **Raw p-value** | **Cohen's d** | **Corrected p-value (X7)** |
| --- | --- | --- | --- | --- | --- | --- |
| Visual N-back | **FP** | 1.41 | 7.25 | 0 | 2.29 | **0.0002** |
|  | **DAN** | 1.58 | 10.34 | 0 | 3.27 | **0** |
|  | **CO** | 1.05 | 6.77 | 0 | 2.14 | **0.0003** |
|  | DN-A | 0.18 | 0.47 | 0.326 | 0.15 | 1 |
|  | DN-B | -0.76 | -4.26 | 0.9989 | -1.35 | 1 |
|  | LANG | 0.47 | 2.16 | 0.0297 | 0.68 | 0.2081 |
|  | **SAL/PMN** | 1.03 | 4.98 | 0.0004 | 1.57 | **0.0027** |
| Auditory N-back | **FP** | 1.55 | 9.5 | 0 | 3 | **0** |
|  | **DAN** | 1.55 | 5.05 | 0.0003 | 1.6 | **0.0024** |
|  | **CO** | 0.93 | 4.15 | 0.0013 | 1.31 | **0.0088** |
|  | DN-A | -0.42 | -1.2 | 0.8692 | -0.38 | 1 |
|  | DN-B | -1 | -6.46 | 0.9999 | -2.04 | 1 |
|  | **LANG** | 1.2 | 3.79 | 0.0021 | 1.2 | **0.015** |
|  | SAL/PMN | 0.34 | 1.24 | 0.1226 | 0.39 | 0.8582 |
| Spatial WM | **FP** | 2.81 | 6.31 | 0.0001 | 1.99 | **0.0005** |
|  | **DAN** | 2.78 | 6.74 | 0 | 2.13 | **0.0003** |
|  | CO | 0.61 | 2.36 | 0.0212 | 0.75 | 0.1483 |
|  | DN-A | 0.91 | 1.56 | 0.0765 | 0.49 | 0.5356 |
|  | DN-B | -0.28 | -0.64 | 0.732 | -0.2 | 1 |
|  | LANG | 0.2 | 0.6 | 0.2803 | 0.19 | 1 |
|  | **SAL/PMN** | 1.64 | 4.53 | 0.0007 | 1.43 | **0.005** |
| Verbal WM | **FP** | 2.27 | 9.11 | 0 | 2.88 | **0** |
|  | **DAN** | 2 | 5.41 | 0.0002 | 1.71 | **0.0015** |
|  | CO | 0.67 | 1.96 | 0.0407 | 0.62 | 0.2849 |
|  | DN-A | 0.17 | 0.4 | 0.3504 | 0.13 | 1 |
|  | DN-B | -0.7 | -3.46 | 0.9964 | -1.09 | 1 |
|  | **LANG** | 1.14 | 5.24 | 0.0003 | 1.66 | **0.0019** |
|  | SAL/PMN | 1.19 | 2.4 | 0.02 | 0.76 | 0.1402 |
| MSIT | FP | 0.31 | 1.02 | 0.1667 | 0.32 | 1 |
|  | **DAN** | 1.68 | 4.3 | 0.001 | 1.36 | **0.007** |
|  | CO | 0.55 | 1.79 | 0.0537 | 0.57 | 0.3757 |
|  | DN-A | -1.23 | -4.27 | 0.999 | -1.35 | 1 |
|  | DN-B | -1.39 | -6.76 | 1 | -2.14 | 1 |
|  | LANG | -0.24 | -1.18 | 0.8652 | -0.37 | 1 |
|  | SAL/PMN | -0.24 | -0.73 | 0.7591 | -0.23 | 1 |
| vMSIT | **FP** | 1.09 | 4.32 | 0.001 | 1.37 | **0.0068** |
|  | **DAN** | 2.68 | 13.76 | 0 | 4.35 | **0** |
|  | **CO** | 1.1 | 7.96 | 0 | 2.52 | **0.0001** |
|  | DN-A | -1.15 | -2.45 | 0.9817 | -0.78 | 1 |
|  | DN-B | -1.8 | -5.9 | 0.9999 | -1.87 | 1 |
|  | LANG | 0.15 | 0.44 | 0.3352 | 0.14 | 1 |
|  | SAL/PMN | -0.35 | -1.05 | 0.8395 | -0.33 | 1 |
| Visual Attention | FP | 0.47 | 1.41 | 0.0954 | 0.45 | 0.668 |
|  | **DAN** | 0.94 | 3.64 | 0.0027 | 1.15 | **0.0189** |
|  | CO | 0.26 | 0.94 | 0.1857 | 0.3 | 1 |
|  | DN-A | -0.65 | -2.17 | 0.9711 | -0.69 | 1 |
|  | DN-B | -0.79 | -4.66 | 0.9994 | -1.47 | 1 |
|  | LANG | -0.24 | -0.74 | 0.7606 | -0.23 | 1 |
|  | SAL/PMN | -0.25 | -0.68 | 0.7445 | -0.22 | 1 |
| Auditory Attention | FP | 0.98 | 2.94 | 0.0083 | 0.93 | 0.058 |
|  | **DAN** | 1.37 | 6.45 | 0.0001 | 2.04 | **0.0004** |
|  | **CO** | 1.31 | 5.97 | 0.0001 | 1.89 | **0.0007** |
|  | DN-A | -0.67 | -2.82 | 0.99 | -0.89 | 1 |
|  | DN-B | -1.17 | -6.44 | 0.9999 | -2.04 | 1 |
|  | **LANG** | 1.55 | 3.57 | 0.003 | 1.13 | **0.0211** |
|  | SAL/PMN | 0.09 | 0.25 | 0.4046 | 0.08 | 1 |

**Supplementary Table S3: Corrected and uncorrected p-values, t-stats and Cohen’s d for all LPFC network task activations to individual cognitive control tasks.** Bolded corrected p-values are significant (corrected p < 0.05).
